# Evaluation of the characterization of acoustic emission of brittle rocks from the experiment to numerical simulation

**DOI:** 10.1038/s41598-021-03910-8

**Published:** 2022-01-11

**Authors:** Fengchang Bu, Lei Xue, Mengyang Zhai, Xiaolin Huang, Jinyu Dong, Ning Liang, Chao Xu

**Affiliations:** 1 Key Laboratory of Shale Gas and Geoengineering, Institute of Geology and Geophysics, Chinese Academy of Sciences, Beijing, 100029 China; 2 Innovation Academy for Earth Science, Chinese Academy of Sciences, Beijing, 100029 China; 3 College of Earth and Planetary Sciences, University of Chinese Academy of Sciences, Beijing, 100049 China; 4 Research Institute of Geotechnical Engineering and Hydraulic Structure, North China University of Water Resources and Electric Power, Zhengzhou, 450046 China

**Keywords:** Solid Earth sciences, Natural hazards

## Abstract

Acoustic emission (AE) characterization is an effective technique to indirectly capture the failure process of quasi brittle rock. In previous studies, both experiments and numerical simulations were adopted to investigate the AE characteristics of rocks. However, as the most popular numerical model, the moment tensor model (MTM) cannot be constrained by the experimental result because there is a gap between MTM and experiments in principle, signal processing and energy analysis. In this paper, we developed a particle-velocity-based model (PVBM) that enabled direct monitoring and analysis of the particle velocity in the numerical model and had good robustness. The PVBM imitated the actual experiment and could fill in gaps between the experiment and MTM. AE experiments of marine shale under uniaxial compression were carried out, and the results were simulated by MTM. In general, the variation trend of the experimental result could be presented by MTM. Nevertheless, the magnitudes of AE parameters by MTM presented notable differences of more than several orders of magnitude compared with those by the experiment. We sequentially used PVBM as a proxy to analyse these discrepancies and systematically evaluate the AE characterization of rocks from the experiment to numerical simulation, considering the influence of wave reflection, energy geometrical diffusion, viscous attenuation, particle size and progressive deterioration of rock material. The combination of MTM and PVBM could reasonably and accurately acquire AE characteristics of the actual AE experiment of rocks by making full use of their respective advantages.

## Introduction

The failure of quasi brittle rock usually experiences crack initiation, crack propagation and crack coalescence in turn^1,2^. By monitoring and modelling this process, engineers can protect and prevent rock mass engineering disasters such as slope instability and rock bursts^3–9^. However, it is always difficult to directly observe the failure process for practical applications. Fortunately, the acoustic emission (AE) phenomenon accompanies the fracturing event, of which characteristics can indirectly reflect the failure process of the rocks^10–13^. Hence, it is essential to capture the AE characteristics of rocks for the monitoring and warning of rock mass engineering disasters^14–17^.

**Figure 1:**
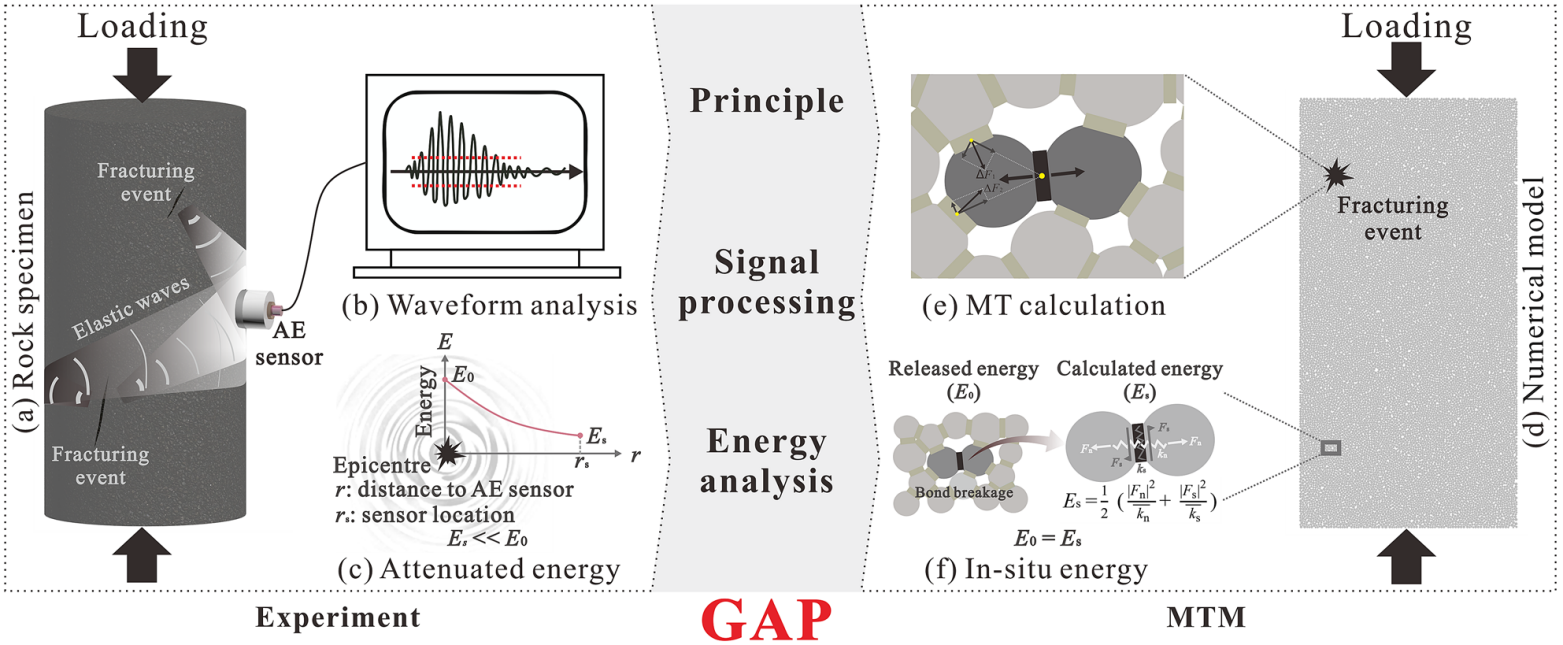
Schematic view of AE characterization by the experiment and MTM.

Laboratory tests have been widely used to investigate the AE characteristics of rocks subjected to external loads. Elastic waves radiated from the fracturing event were recorded by the piezoelectric sensor attached to the surface of the rock sample, as shown in Fig. 1a. Then, these signals were amplified by a preamplifier and denoised by a filter. Then, AE signals were further amplified by a main amplifier and quantitively analysed to determine the digitized waveform data in the form of time range, including AE events, energy release and the *b*-value, which presented the failure process of rocks. However, there remained two main limitations to the experimental method^18^. On the one hand, only when the AE signal propagated to the sample surface could it be monitored by piezoelectric sensors. During this process, the radiated energy was attenuated due to both geometrical diffusion and viscous dissipation. The monitored signals thus could not completely represent epicentre cases. On the other hand, the monitored waves were superimposed waves resulting from multiple wave reflections at interfaces, which posed challenges in recognizing a series of independent AE events. Meanwhile, the failure of the stressed rocks could be represented only as AE signals monitored by a limited number of sensors. Additionally, the fracturing process inside the rock sample could not be visible in real time to calibrate the AE signals^19^.

Out of the experiment, numerical models were also applied, which had the capability of simultaneously characterizing the rock failure and AE behaviours. Via the finite element method (FEM), the fracturing behaviour is usually achieved by degrading material properties according to continuum laws, and a damaged element is regarded as an AE event, which was reflected in a typical code called RFPA proposed by Tang^20^. Based on a similar principle, researchers have developed other AE simulation models, such as the local linear tensorial damage model^21^, local degradation model^22^ and elastoplastic cellular automata model^23^. Using the discrete element method (DEM), a rock material is expressed in the form of discrete elements connected by contact bonds. The DEM makes quasi-static deformation simulation possible by solving a motion equation and has the advantages of explicitly accomplishing a fracturing process in rocks and simulating elastodynamic effects of stress wave propagation and cracking-induced AE^24–26^. Typically, Hazzard and Young^24^ proposed a dynamic AE recording technique by directly quantifying the kinetic energy of particles into the energy radiated by seismic sources when bonds are broken in a 2D particle-based model. Then, this technique was improved by introducing moment tensor calculation by tracking the change in contact force at the time bonds broke, which is referred to as the moment tensor model (MTM)^27^. In addition to the FEM and DEM, there are other AE simulation models, such as the static lattice model^28^, continuum fracture mechanics model^29^, quasidynamic monitoring kinetic energy model^30,31^, deviatoric strain rate model^32^ and Voronoi element-based explicit numerical manifold model^33^. Among the aforementioned models, MTM based on DEM has been widely used in simulating AE benefiting from the ability of DEM to explicitly represent fractures and bond failure of rocks and excellent applicability of MTM to quantitatively characterize AE^34–37^.

On the above basis, many researchers have attempted to explore the AE characteristics of rocks from experiments to MTM directly. Ma et al.^38^ acquired different numbers of AE events compared with experimental results by reproducing Brazilian tests using MTM. Chorney et al.^39^ compared MTM with experiments in AE energy by simulating triaxial compression tests of sandstone. Zhang and Zhang^40^ investigated the difference in the relative magnitude of *b*-value drop-offs between experiments and MTM by modelling uniaxial compression tests of limestone. However, it is not convincing to directly compare simulated AE characteristics with those in experiments due to some key contradictions between MTM and the actual AE test^41^, as shown in Fig. 1. In terms of the principle, MTM treats bond breakages occurring close in time and space as a single AE event (Fig. 1e), while actual AE tests treat superposed elastic waves that exceed a threshold and cause a system channel to accumulate data (Fig. 1b) as a single AE event^27,42^. In terms of the processing method, MTM realizes quantitative AE characterization by calculating the moment tensor based on the change in contact forces upon particle breakage (Fig. 1e), while actual AE tests quantify AE by analysing superimposed waveforms (Fig. 1b) acquired from AE sensors attached to the surface of specimens^43,44^. In terms of energy analysis, epicentre energy can be explicitly calculated by MTM (Fig. 1f), while the energy acquired by the experiment is just a part of the epicentre energy because of geometrical diffusion and viscous dissipation from the epicentre to the AE sensor location (Fig. 1c). The potential breakable bonds in the numerical model are often far fewer than the potential breakable bonds of the actual rock considering the computational capability, resulting in smaller numbers of AE events by MTM than the experiment, especially for the 2D numerical model.

From the literature review above, the result by MTM cannot be constrained by the experimental result, as it did not reproduce the monitoring and analysis manner of AE signals of the physical experiment. Evaluation of the consistency and compatibility between the experiment and MTM, which is the motivation of this study, is thus important. This paper was structured as follows. “AE characterization by the experiment and MTM” section overviews the MTM and simulates an AE experiment. “Particle-velocity-based model” section introduces the proposed PVBM (particle-velocity-based model) and shows reproduced results using PVBM in detail. These results are discussed in “Discussion”. Conclusions are given in “Conclusions” section.

## AE characterization by the experiment and MTM

### Experiment

**Figure 2:**
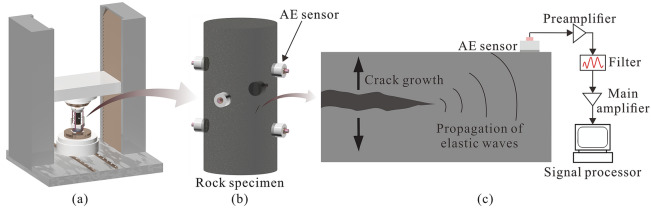
Schematic view of uniaxial compression tests with AE recording. (**a**) Loading system; (**b**) AE monitoring system; and (**c**) AE signal processing.

Three Marine shale cylindrical specimens cored from the Longmaxi Formation in the Pengshui shale gas area in China were used to conduct uniaxial compression tests with AE in a laboratory. As shown in Fig. 2a, the samples were all subjected to unconfined compressive loading to failure with a constant rate of 10^–5^ s^-1^ by the RTR-2000 triaxial dynamic testing system of GCTS (USA). Figure 2b shows the AE monitoring system with six AE sensors mounted on the surface of the specimen by rubber band and tape. The sampling frequency was set as 1 MHz, and the amplitude threshold was set as 35 dB^10,45^. The quantitative analysis of AE data was based on the specific AE sensor that collected the longest dataset length of AE events^9^. Actual AE parameters, including events and energy, were acquired by this system. Considering that the *b*-value represents the scale of the magnitude distribution of AE and has been widely used to characterize the scale of crack growth, it was calculated using the Gutenberg–Richter formula^46^.

**Figure 3:**
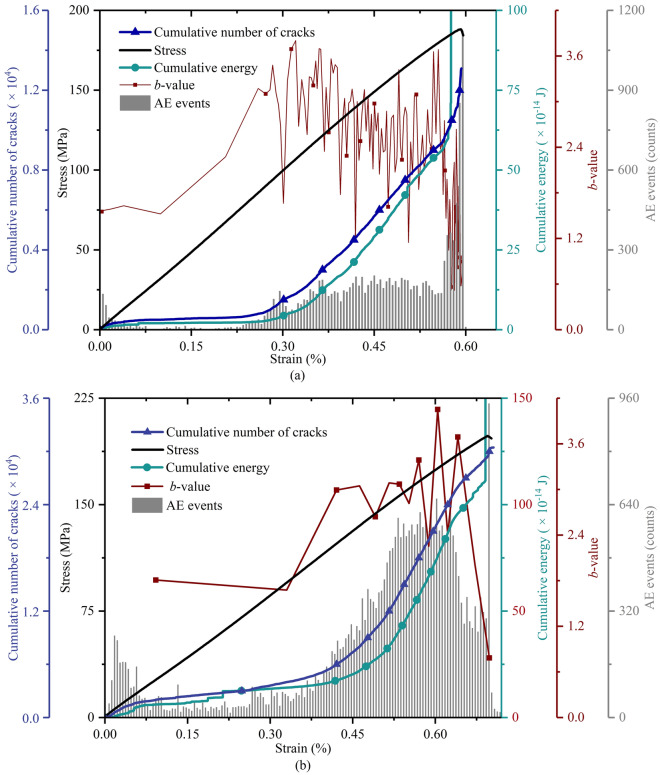
Axial stress, cumulative number of cracks and AE parameters (cumulative energy, *b*-value and AE events) versus the axial strain by (**a**) experiment #1; and (**b**) experiment #2.

**Figure 4:**
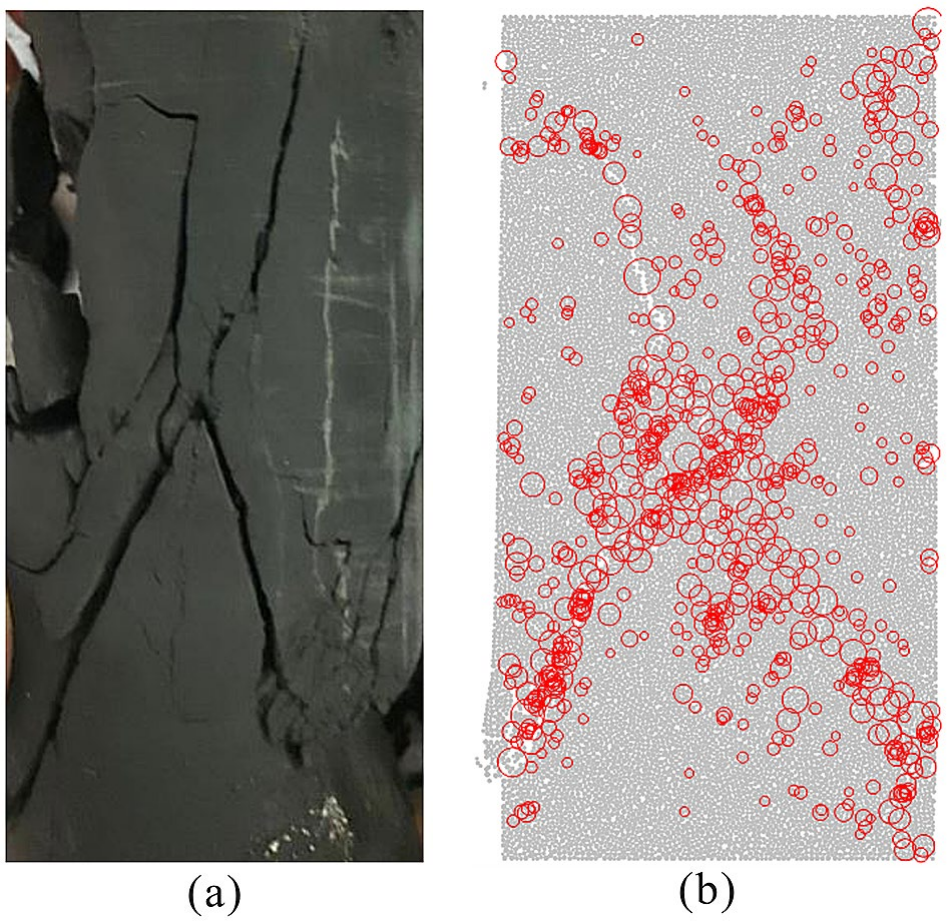
Calibration of the failure configuration between (**a**) experiment #2; and (**b**) simulation. The red circles correspond to simulated AE events, and the size of the circle represents the local magnitude.

Figure 3 shows the variation in axial stress, cumulative number of cracks and actual AE parameters (cumulative energy, *b*-value and AE events) versus axial strain of experiment #1 and #2. Although properties of both specimens were not definitely consistent in number, they showed similar variation trends. Experiment #2 was taken as an example to analyse the experimental AE results. At the beginning of loading (strain from 0 to 0.06%), the stress–strain relationship exhibits an approximately linear manner. Note that the curves of cumulative energy and cracks increase slightly with increasing strain, which corresponds to some AE events. The *b*-value is initially 1.81. The above values may contain inevitable deviations. The compaction of natural cracks in the shale specimen or the rupture of small bulges at the ends of the specimen may cause some false AE events^47^, which directly affects the calculation of cracks because the cumulative number of cracks has been widely claimed to be calculated by adding up the number of AE events. As the strain ranges from 0.06% to 0.26%, the stress curve increases almost linearly, while the curves of cumulative energy and cracks hardly increase, involving few AE events. At this stage, the *b*-value decreases slightly. These phenomena show that the specimen nearly produced a linear elastic response at this stage. When the strain falls in the range (0.26%–0.69%), the stress curve increases nonlinearly, and the curves of cumulative energy and cracks increase greatly with respect to the approximately linear growth of AE events. The *b*-value curve fluctuates and exhibits a sudden drop when the strain reaches 0.67%. Interestingly, AE events increase approximately linearly with strain ranging from 0.33% to 0.52%, out of which it fluctuates at 600 until a strain of 0.62%. Then, AE events drop sharply, and the variation rate of cumulative energy and cracks decreases, suggesting that the rock might experience a transition from stable crack growth to unstable growth. Finally, when the strain is over 0.69%, the stress curve lies in postpeak stages, and the curve of cumulative energy increases strongly, corresponding to the strong increase in AE events. The *b*-value curve drops significantly, which indicates that the specimen reaches peak strength (unconfined compression strength, UCS), and the cracks coalesce with each other on a large scale. Sequentially, the whole specimen was fractured into pieces, as shown in Fig. 4a.

### MTM

#### Theory of MTM

The MTM algorithm is implemented based on the particle flow code (PFC) of the DEM developed by the ITASCA Consulting Group (USA), in which a rock material is modelled as an assembly of bonded particles^24,48^. When external loads exceed the bond strength between two particles, they will be broken and release stored energy, causing movement of particles on both sides and deformation of adjacent contact bonds^24–27^. These changes can be quantified by calculating the components of the moment tensor by summing the force change at the contact multiplied by the distance of the contact from the bond breakage location^27^:

1 $$M_{{{\text{ij}}}} = \mathop \sum \nolimits_{k = 1}^{N} F_{i}^{K} \times R_{j}^{k}$$

where $M_{{{\text{ij}}}}$ is the value of the moment tensor, *N* is the number of particles involved in making an event, $F_{i}^{K}$ is the *i*th component of the unbalanced force concentrated at the centre of the *K*th particle, and $R_{j}^{k}$ is the *j*th component of the distance between the contact point and the centre of the *K*th particle. MTM ingeniously complies with this principle by monitoring all bond breakages during a simulation and treating bond breakages occurring close in time and space as a single AE event. To improve the operation efficiency, the single moment tensor of each AE event is the maximum scalartensor, which can be determined by the eigenvalue of the moment tensor matrix (*m*_j_):

2 $$M_{0} = \left( {\frac{{\mathop \sum \nolimits_{j = 1}^{3} m_{j}^{2} }}{2}} \right)^{\frac{1}{2}}$$

The moment magnitude of the AE event (*M*_w_) can be determined with an empirical Eq. ^49^:

3 $$M_{{\text{w}}} = \frac{2}{3}{\text{log}}M_{0} - 6$$

Compared with the moment magnitude information, AE energy and *b*-value are more widely used to depict AE characteristics. The relationship between magnitude (*M*_w_) and AE energy has been proven to be able to be expressed by the empirical formula Eq. (4):^50^

4 $${\text{Log}}E = 1.5 \times M_{{\text{w}}} + 4.8$$

and the AE amplitude distribution conforms to the Gutenberg–Richter relationship:

5 $${\text{Log}}N = a - b \times M_{{\text{w}}}$$

where *a* and *b* are constants, and *N* is the number of simulated magnitudes that exceed *M*_w_.

#### AE analysis by MTM

PFC^2D^ was adopted to simulate the above experiment. The numerical model has a width of 50 mm and height of 100 mm and is made up of 16,884 particles with radii uniformly ranging from 0.21 mm to 0.35 mm. The parallel-bond model and smooth joint model were applied to describe the mechanical response of the grain boundaries and joints of the actual shale specimen when loaded^51^. Upper and lower boundaries were applied to the relative velocity of ± 0.05 m/s considering the amount of calculation^52^. Usually, the macroscale mechanical behaviours of rocks relate to the microproperties. In the calibration process, multiparameter sensitivity analysis was adopted to determine the default initial microparameters and reasonable ranges^53^. Next, according to constraints given by experimental results, parameters and ranges were further determined. Finally, these parameters were calibrated based on previous macromicroscopic parameter relationships until the simulated results were basically in line with the experimental results^32,44,53^. The microproperties in Table 1 have been calibrated for a range of UCSs and Young’s moduli based on the data presented in Table 2. Figure 4 shows the calibrations of the failure configuration, indicating that the simulation agreed well with the experimental results. Simulated AE parameters, including AE event, energy and *b*-values, were acquired by MTM.

**Table 1:** Calibrated microproperties used in PFC^2D^ to present the marine shale specimen.

Particle parameters		Parallel bond parameters	
Density (kg/m^3^)	3000	Bond effective modulus (GPa)	22
Effective modulus (GPa)	22	Bond stiffness ratio	1.5
Stiffness ratio	1.5	Tensile strength (MPa)	170
Friction coefficient	0.8	Cohesion (MPa)	150
Damping coefficient	0.7	Friction angle (°)	40
Smooth joint parameters			
Normal stiffness (GPa/m)	10,000	Tensile strength (MPa)	30
Shear stiffness (GPa/m)	3700	Cohesion (MPa)	80
Friction coefficient	20	Joint friction angle (°)	40

**Table 2:** Calibrated results of macro properties.

	Experiment			Simulation
	1	2	3	
UCS (MPa)	188.16	198.42	166.67	173.12
Young’ s modulus (GPa)	32.34	27.37	23.78	27.59

**Figure 5:**
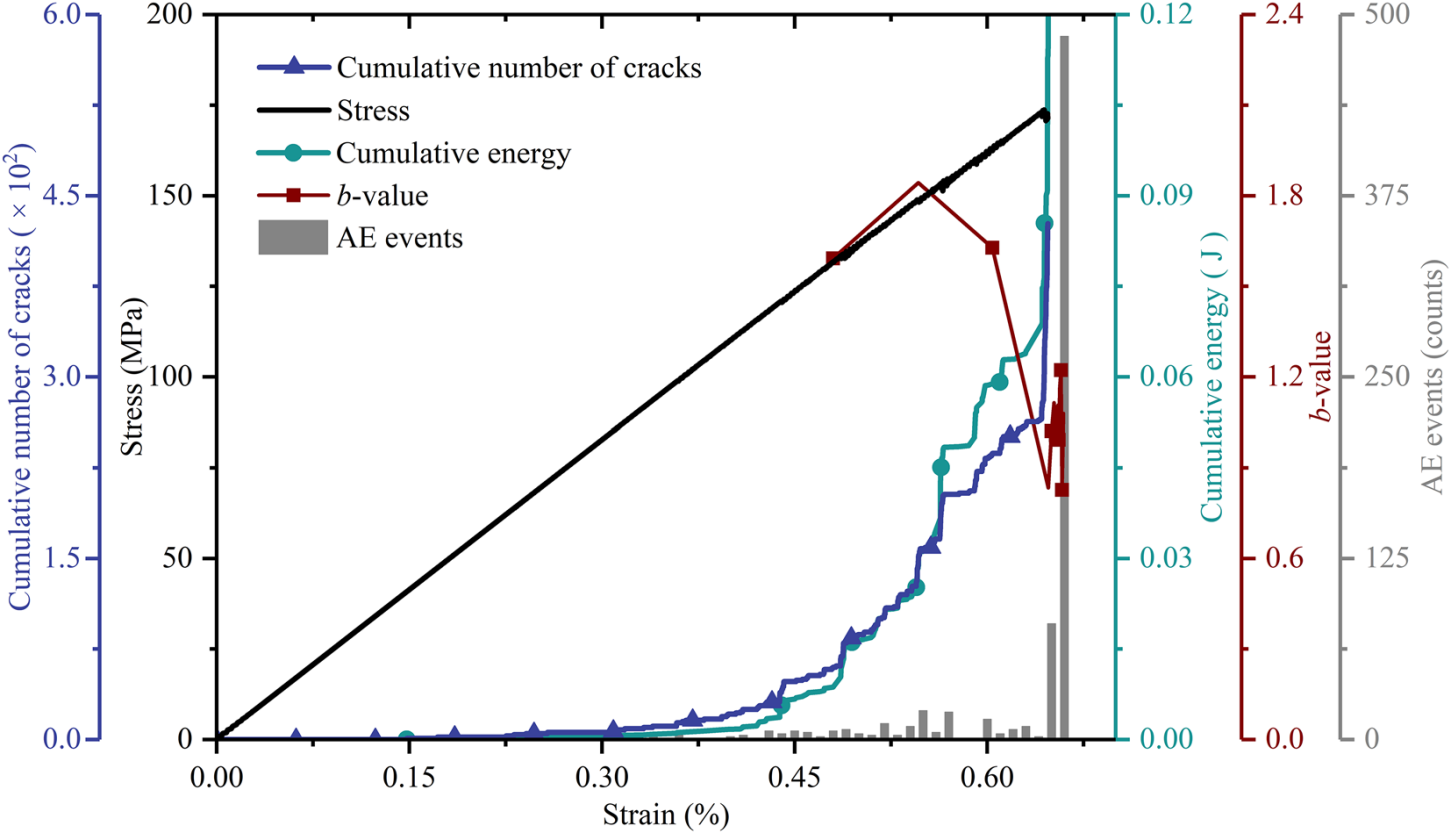
Axial stress, cumulative number of cracks and simulated AE parameters (cumulative energy, *b*-value and AE events) versus axial strain by MTM.

Figure 5 shows the AE characteristics simulated by the MTM. During loading with strains ranging from 0 to 0.40%, the stress–strain relationship shows a linear manner, while the curves of cumulative energy and cracks remain basically invariant, which corresponds to few AE events. These phenomena show that the synthetic rock model displays elastic properties over this period. Then (strain from 0.40% to 0.65%), the stress curve fluctuates slightly, while both curves of cumulative energy and cracks increase in the form of a stepwise mode, which is involved in distinct AE events. Finally, when the strain is over 0.65%, the stress–strain curve experiences the postpeak stage, and the curves of cumulative energy and cracks increase from 0.0687 J to 2.5105 J and from 278 to 427 in number, the corresponding *b*-value decreases from 1.06 to 0.83. These phenomena indicate that the numerical model reaches the peak strength point.

### Comparison of AE characterization between MTM and the experiment

Clearly, AE characterization by MTM displays variation trends similar to the experimental results: At first, there are few AE events and invariability of energy and cracks, then AE events increase, and both curves of cumulative energy and cracks increase greatly, finally AE events and curves of cumulative energy and cracks all occur in breakthrough at the peak strength point, corresponding to the sharp decrease of *b*-values.

However, some discrepancies in AE characterization are remarkable. For the number of AE events, the experiment records 30,502, while the MTM records 734. In addition, at the beginning of loading, there are some AE events by the experiment but not by the MTM since AE events caused by pre-existing defects, such as natural cracks, are difficult to reproduce in the simulation^54^. AE events during the prepeak stage are not appreciable by MTM (Fig. 5). For the energy, comparisons show that energy by MTM is more than 10 orders of magnitude more than the energy by the experiment. For the *b*-value, the simulated *b*-value acquired by the MTM varies over a smaller range than the *b*-value acquired by the experiment. It is difficult to directly explain the abovementioned discrepancies in AE characterization because there is a gap between MTM and experiments in principle, signal processing and energy analysis, as illustrated in Fig. 1.

## Particle-velocity-based model

### Implementation

To fill in the gap between the experiment and MTM, a proxy model was proposed to imitate the experimental process, and the model was compared with MTM. The proxy model used the particle velocity monitored to analyse the AE characteristics of the numerical model, named the particle-velocity-based model (PVBM). Although previous studies^31,55^ have used element velocity to represent kinetic energy, to the best of our knowledge, no attempt has been made yet to describe AE characteristics by PVBM.

**Figure 6:**
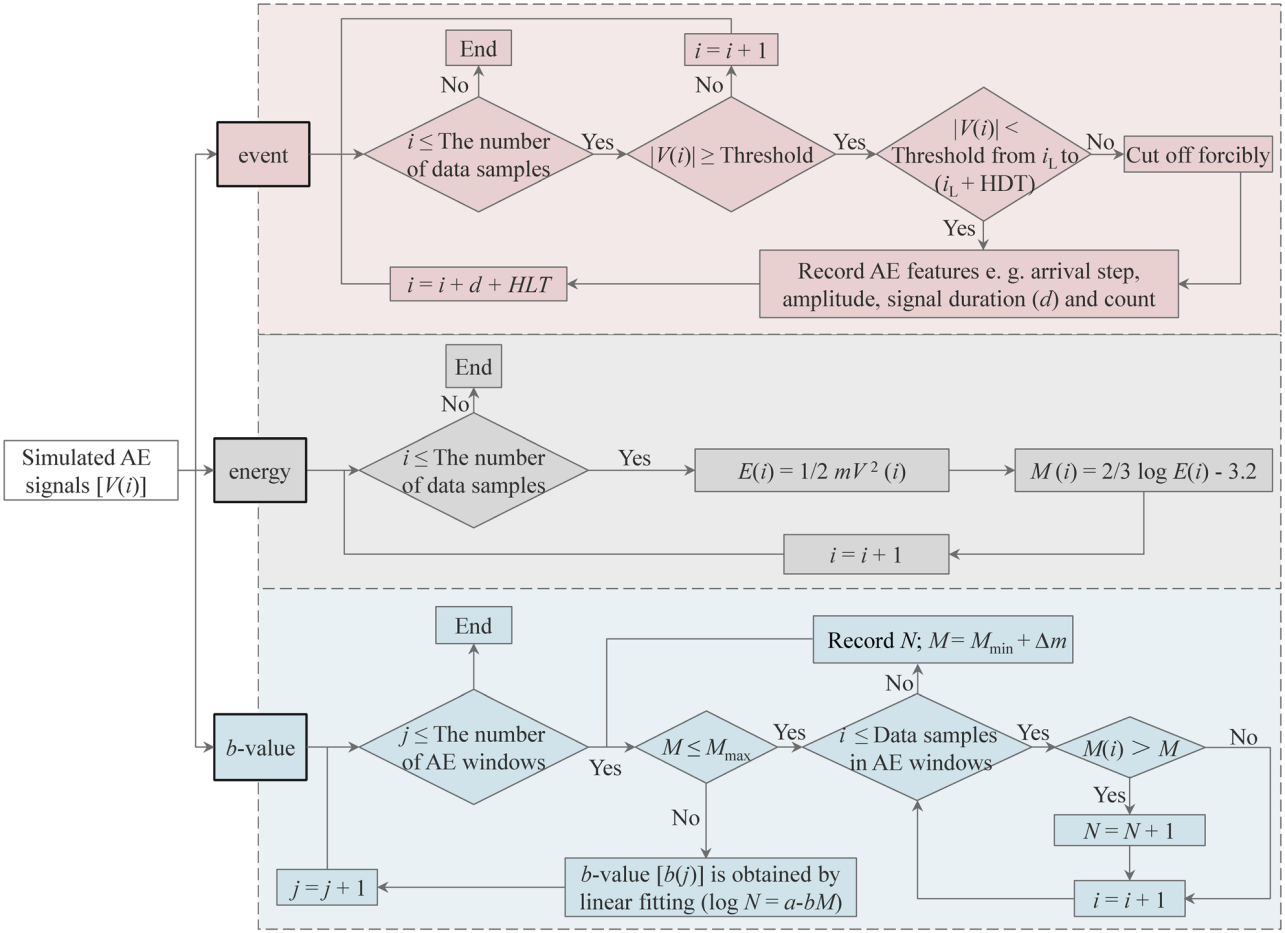
Flow chart of add-in code to calculate simulated AE characteristics. The simulated AE signal is represented by the normal velocity component *V*(*i*), which is imported into three calculation modules to obtain the event, energy and *b*-value, where *i* is the step rather than time, *i*_L_ is the last threshold crossing step, *d* is the signal duration, HDT is the hit definition time, HLT is the hit lockout time, *E*(*i*) is the AE energy, *m* is the particle mass, *M*(*i*) is the magnitude, ∆*m* is the magnitude interval, *j* is the number of AE windows, and *N* is the number of simulated magnitudes that exceed (*M* + ∆*m*).

As shown in Fig. 2c, the fracturing of the rock material is accompanied by the release of stored strain energy in the form of elastic waves. For the actual AE test, these elastic waves propagate inside the medium and can then be detected by AE sensors attached to the sample^56^. The PVBM complies with the rules of the experiment at most, that is, simulated AE signals are acquired by monitoring the normal particle velocity on the surface of a numerical model at each step in PFC^2D^. The next step is to analyse the simulated AE signals. In an actual AE test, AE signals are analysed by a signal processor in the form of waveform analysis, of which a typical commercial software is AEwin developed by Physical Acoustics Corporation (USA). However, the simulated AE signals cannot be analysed directly by AEwin because the time evolution of PFC^2D^ is computed via the step^48^. Therefore, a series of add-in codes was developed by combining the principle of AEwin and the calculation characteristics of PFC^2D^. Finally, we acquired simulated AE characteristics of a rock model, mainly including event, energy and *b*-value, which were designed in the following context (Fig. 6).

For the simulated event, one or more predetermined evaluation thresholds should be set to identify simulated AE signals. When |*V*(*i*)| exceeds the threshold, it marks the beginning of a simulated AE event, and *i* is determined as an arrival step. As the step increases, the last threshold crossing is recorded as *V*(*i*_L_). Next, judge whether this simulated event is over. The hit definition time (HDT) and Max duration are used to achieve this goal. If the threshold is not exceeded by |*V*(*i*)| from *i*_L_ to (*i*_L_ + HDT), it marks the end. The other case is that a simulated AE signal will be cut off forcibly when its length reaches the Max duration, which is often used in the acquisition of continuous signals or the stage of very intense signals. After confirming the end of a simulated event, other simulated AE features, such as signal duration (*d*) and amplitude, are recorded. To prevent reflections of the former signal from being taken as a start of the next signal, hit lockout time (HLT) is defined. Finally, *V*(*i* + *d* + HLT) will return to judge the next simulated event until all data have been calculated.

For the simulated energy, the kinetic energy of particles can be determined explicitly in the DEM. The simulated *b*-value can be calculated by Eq. (5). Reasonable magnitude range [*M*_min_, *M*_max_] and AE windows, i.e., AE event segmentation should be predetermined to achieve this linear fitting. For data samples *V*(1) ~ *V*(*i*) in the *j*th AE windows, *N* is the number of simulated magnitudes that exceed (*M* + ∆*m*), where ∆*m* is the magnitude interval. Then, a set of correlation data between *N* and *M* is obtained to calculate *b*(*j*) by Eq. (5) fitting with a least square method. Then, *b*(*j* + 1) will be calculated until all AE windows are completed.

### AE characterization by PVBM

#### Monitoring points

**Figure 7:**
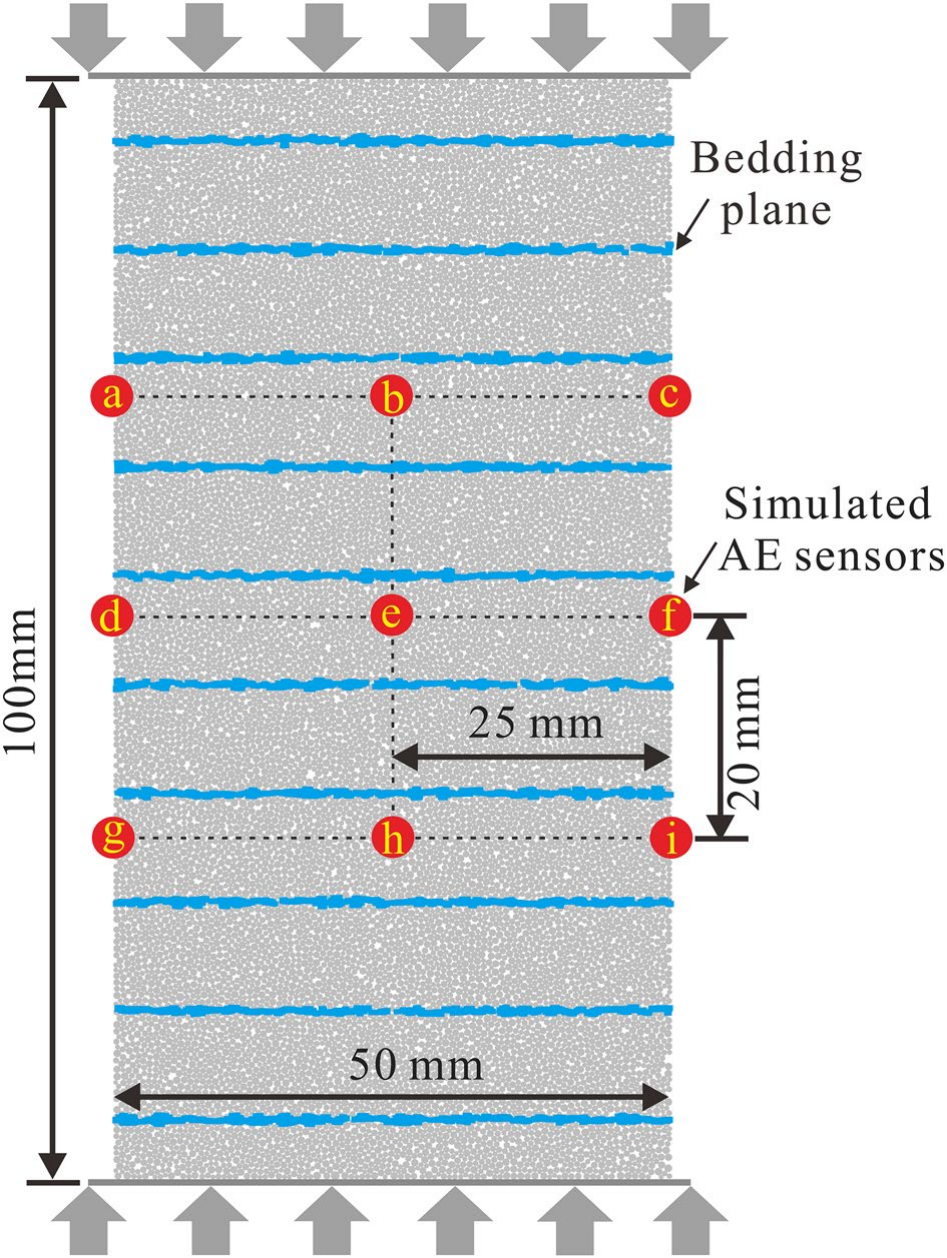
The layout of simulated AE sensors #a ~ #i on the numerical model. The axial and lateral spacing is 20 mm and 25 mm, respectively.

To maintain comparability, the numerical model and boundary conditions were the same as the numerical model and boundary conditions in AE analysis by MTM section except that nine monitoring points #a ~ #i were set, as shown in Fig. 7. Each monitoring point can be regarded as an AE sensor. The points were equidistantly placed in the axial direction and lateral direction with spacings of 20 mm and 25 mm, respectively, to obtain the normal velocity of the monitoring points at each step. Then, the acquired simulated AE signals were imported into the add-in code (Fig. 6) to obtain the final simulated AE parameters. Based on many trials and considering wane and wax, it is recommended that the evaluation threshold value, AE event segmentation, Max duration and HDT be set as 0.05 m/s, 50, 40 steps and 20 steps, respectively.

#### Robustness of PVBM

To evaluate the robustness of the PVBM, nine waveforms of simulated AE sensors #a ~ #i were imported into the add-in code to acquire simulated AE parameters and to be compared.

**Figure 8:**
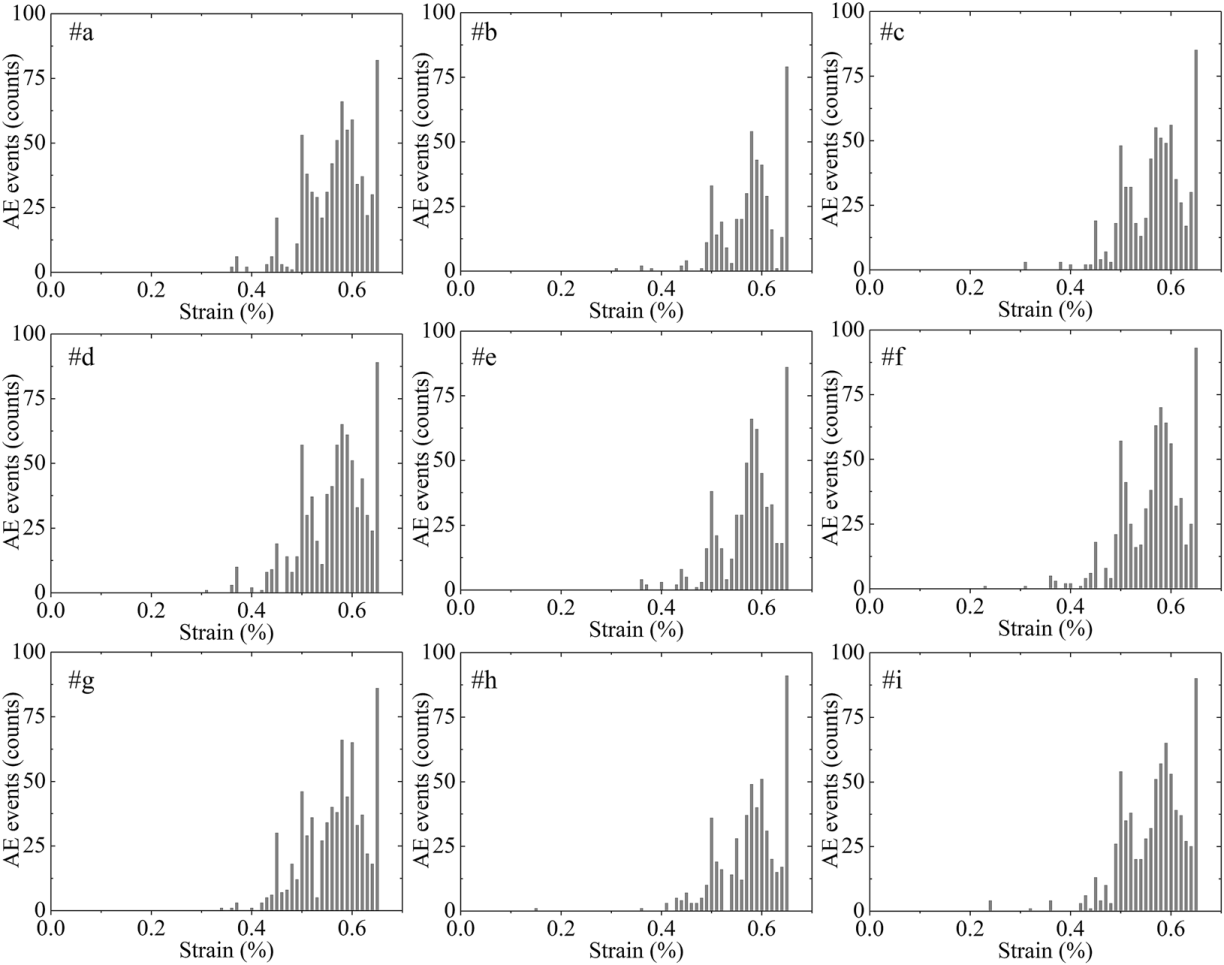
The number of simulated AE events at AE sensors #a ~ #i versus axial strain by PVBM.

Figure 8 shows the variation in simulated AE events at AE sensors #a ~ #i. The results show a similar variation in which the simulated AE events emerge at a strain of approximately 0.36% and experience several isolated peaks at strains of approximately 0.45%, 0.49%, 0.58% and 0.66% with increasing values in a sequence. These similarities show good robustness of AE events by PVBM.

**Figure 9:**
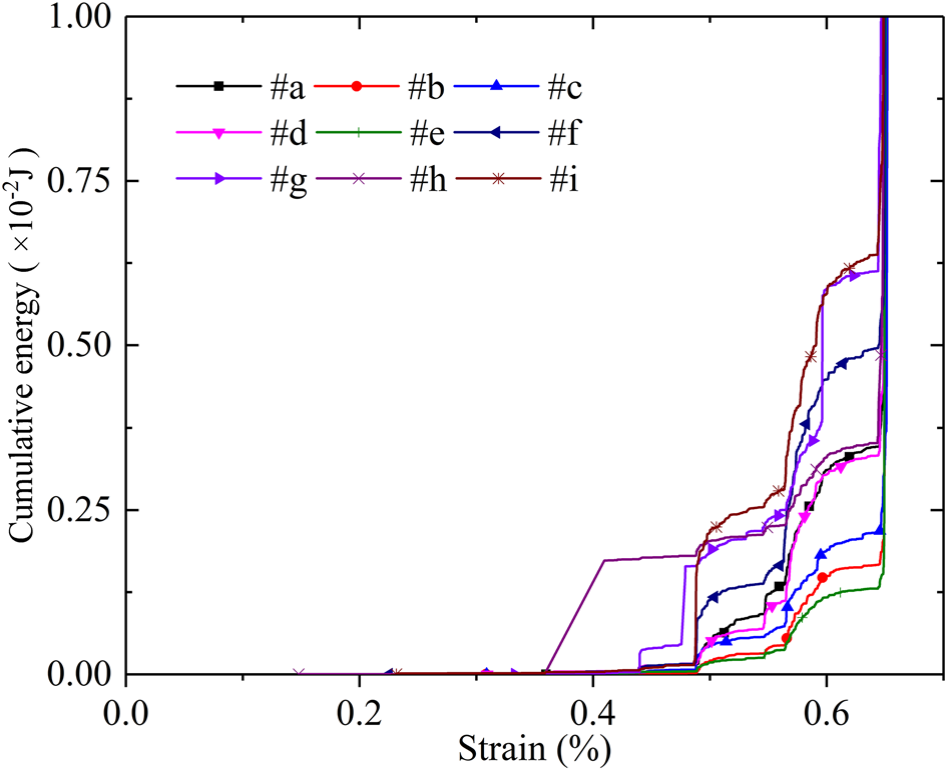
The variation of simulated cumulative energy at AE sensors #a ~ #i versus axial strain by PVBM.

Figure 9 shows the variation of simulated cumulative energy at AE sensors #a ~ #i. The results show a similar variation trend: The simulated cumulative energy increases first at a strain of approximately 0.45%, then increases in the form of a stepwise mode, finally soars at a strain of approximately 0.64%. In this process, the strains corresponding to the thresholds of stepwise growth on the nine curves are almost the same at approximately 0.45%, 0.49%, 0.55% and 0.56%. In general, the identification of thresholds of cumulative AE energy by PVBM shows good robustness. However, the variation magnitudes are slightly different, since some epicentres are away from the receiver, but some are close.

**Figure 10:**
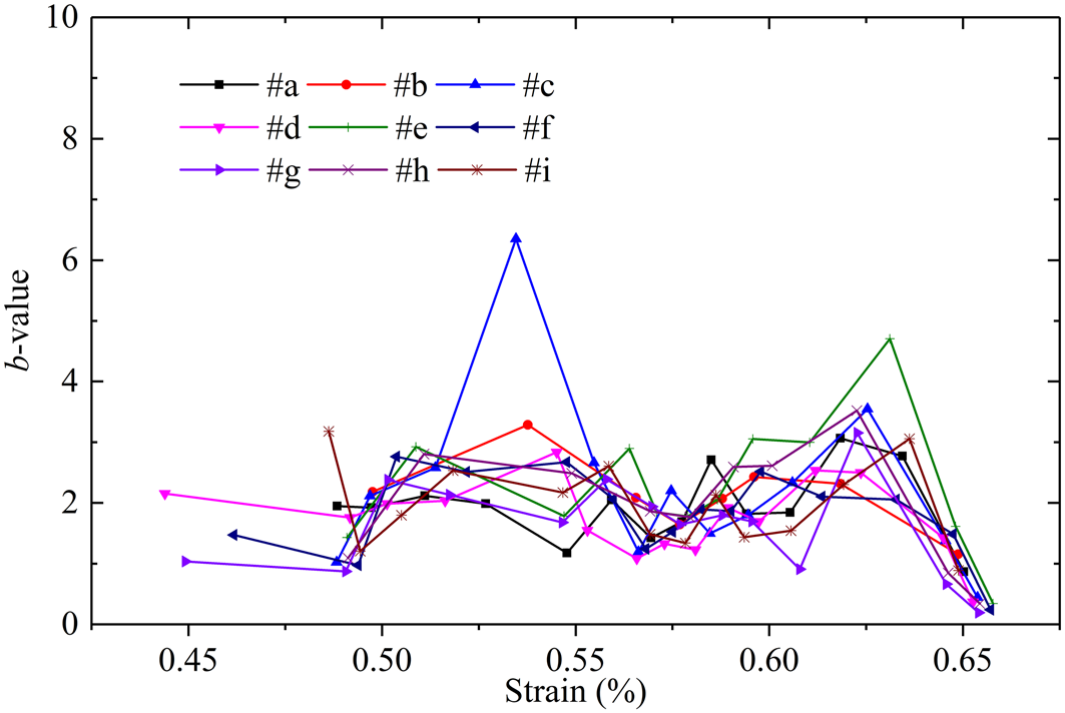
Variation in simulated *b*-value at AE sensors #a ~ #i versus axial strain by PVBM.

Figure 10 shows the variation in the simulated *b*-value at AE sensors #a ~ #i. The trends of the simulated *b*-value display approximate hump curves. The *b*-values all begin to increase adjacent to strains of 0.49% and 0.56%. In contrast, the *b*-values all begin to decrease adjacent to strains of 0.55% and 0.64%, and the latter falls more severely, corresponding to the peak point of the numerical model. Generally, the identification of turning points of *b*-values by PVBM displays good robustness.

**Figure 11:**
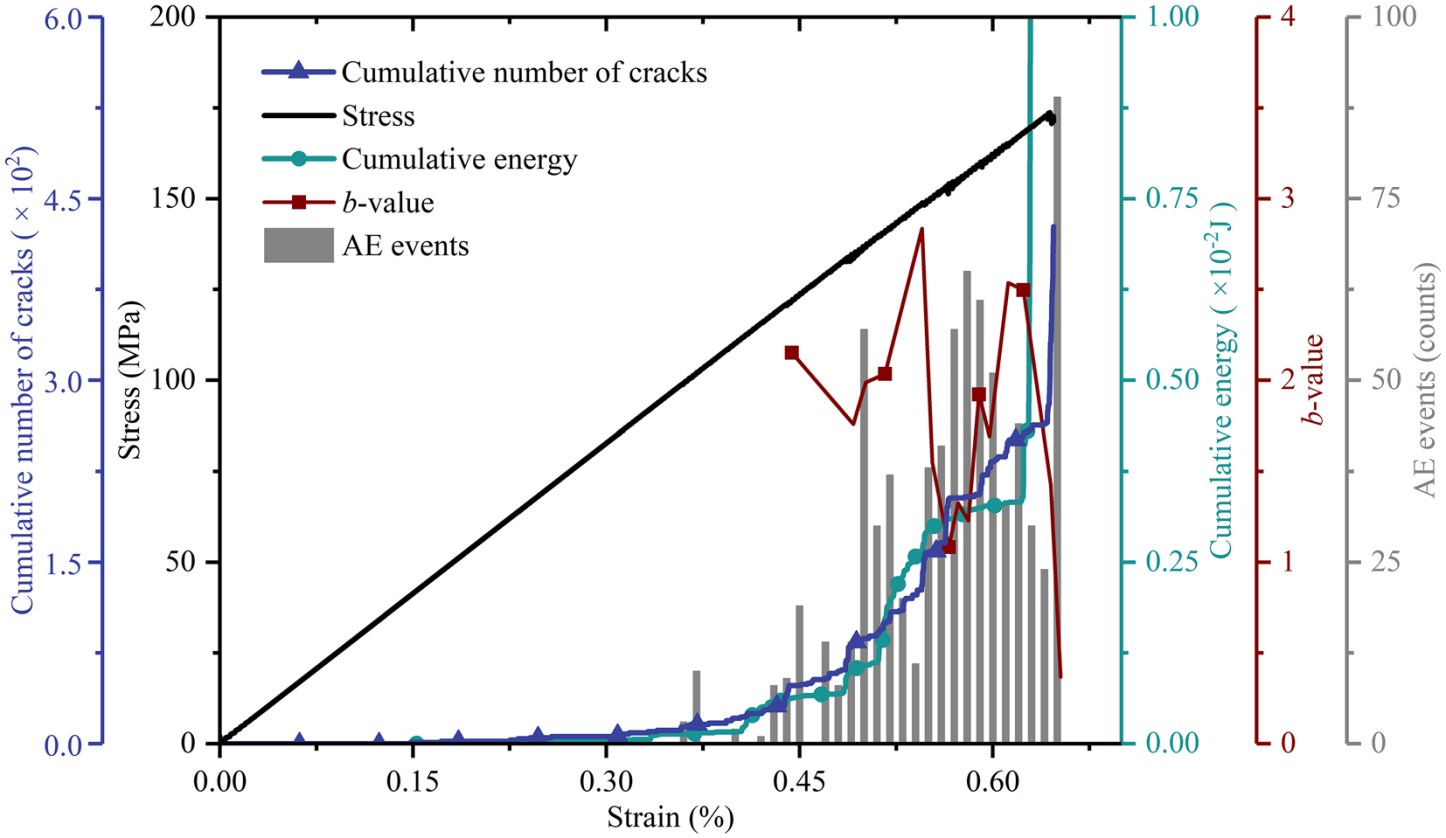
Axial stress, cumulative number of cracks and simulated AE parameters (cumulative energy, *b*-value and AE events) versus axial strain at AE sensor #d by PVBM.

AE characterization by PVBM shows good robustness. We used data from the simulated AE sensor #d for systematic analysis. Considering the consistency of the numerical model and boundary conditions, the curves of stress and crack versus strain by PVBM are the same as the curves of stress and crack versus strain by MTM. As shown in Fig. 11, at first, there are few AE events and approximately invariant energy, then the former increases greatly, and the latter increases in the form of a stepwise mode, while the simulated *b*-value fluctuates in a hump manner. Interestingly, when the stress–strain curve fluctuates perceptibly, AE events synchronously gather into solitary peaks, curves of cumulative energy and cracks concurrently increase in a stepwise manner, and the *b*-value simultaneously decreases, which indicates that there are fracturing events on a large scale at these thresholds, including at strains of approximately 0.45%, 0.55%, 0.59% and 0.65%^38^. The maximum extent is at a strain of approximately 0.65%, where AE events increase from 24 to 89, and the curves of cumulative energy and cracks soar from 0.0034 J to 1.0625 J and from 278 to 427 in number, the corresponding *b*-value decreases from 2.50 to 0.37, indicating that the numerical model reaches the peak point.

## Discussion

AE monitoring and modelling are very important for the prevention of rock mass engineering disasters. However, there are some contradictions between the actual AE test and AE simulation. By PVBM, we investigated these discrepancies and evaluated AE characterization, including AE events, energy and *b*-values of rocks, from the experiment to numerical simulation.

### AE event

**Figure 12:**
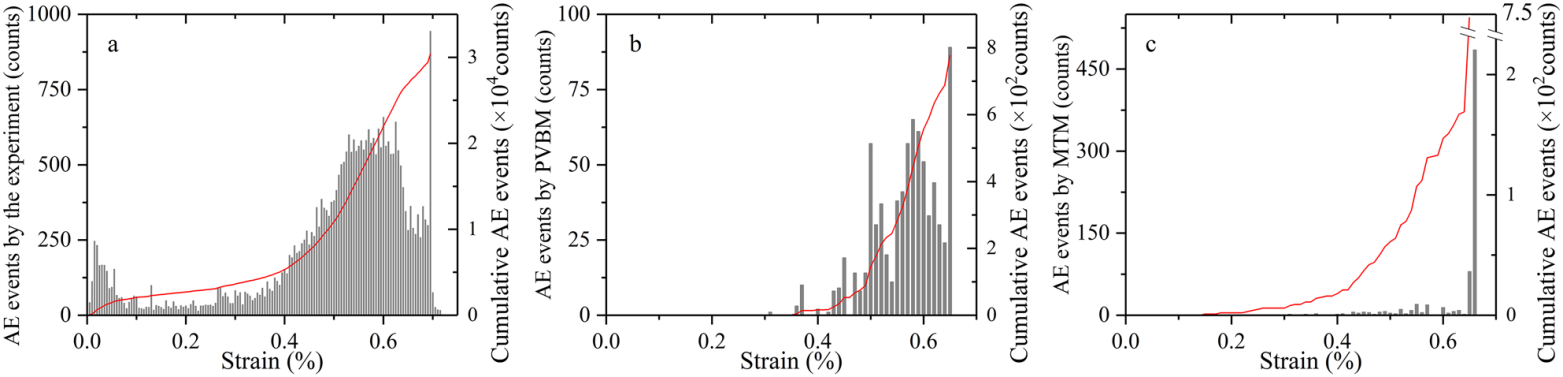
Comparison of AE events by the (**a**) experiment; (**b**) PVBM; and (**c**) MTM.

Figure 12 shows the comparison of AE events. The histograms all show a similar variation trend in which AE events are relatively few at first, then they increase and form approximate solitary peaks before soaring at the peak points. However, this trend by MTM is not appreciable compared with PVBM, manifested by smaller solitary peaks during the prepeak stage and final excessive mutation at the peak point. The number of AE events of the prepeak stage by the MTM is approximately 2.5 times less than the number of AE events of the prepeak stage by the PVBM, while the number of AE events by the MTM at the peak point is approximately 5.45 times more than the AE events by the PVBM at the peak point.

For the difference in the prepeak stage, one possible reason is that in the MTM, given that a newly formed crack is within one particle diameter of an existing crack, and the event is still in the duration determined by assuming that a fracture propagates at half the shear wave velocity of the numerical model, this newly formed crack and the existing crack will be considered the same AE event^57^. The other possible reason is that there are signal reflections at interfaces when using PVBM, which causes the final received simulated AE signal to be a superposition of the wave from the epicentre and all its reflections^58^.

For the difference at the peak point, since PVBM is based on waveform analysis, AE signals are difficult to identify and separate accurately at the peak point, as there are too many waveforms, including the superposition and mutual influence of AE events received in such a short period. These signals are always treated as continuous signals and are cut off forcibly^59^. This difference may be improved by introducing some experimental data processing methods^60^. In addition, the large-scale coalescence has a great influence on the transmission of elastic waves^61^.

AE events can intuitively reflect the failure process of rock. Thus, they have been widely used for the prediction of rock mass engineering disasters. The abovementioned evaluation of AE events indicates that the pattern of AE events by MTM is similar but not appreciable compared with the pattern of AE events by the experiment and PVBM, which hampers the identification of the prepeak fracturing. Fortunately, PVBM covers this shortage. We thus recommend a combination of MTM and PVBM when using numerical simulations to supplementarily predict rock mass engineering disasters.

### AE energy

**Figure 13:**
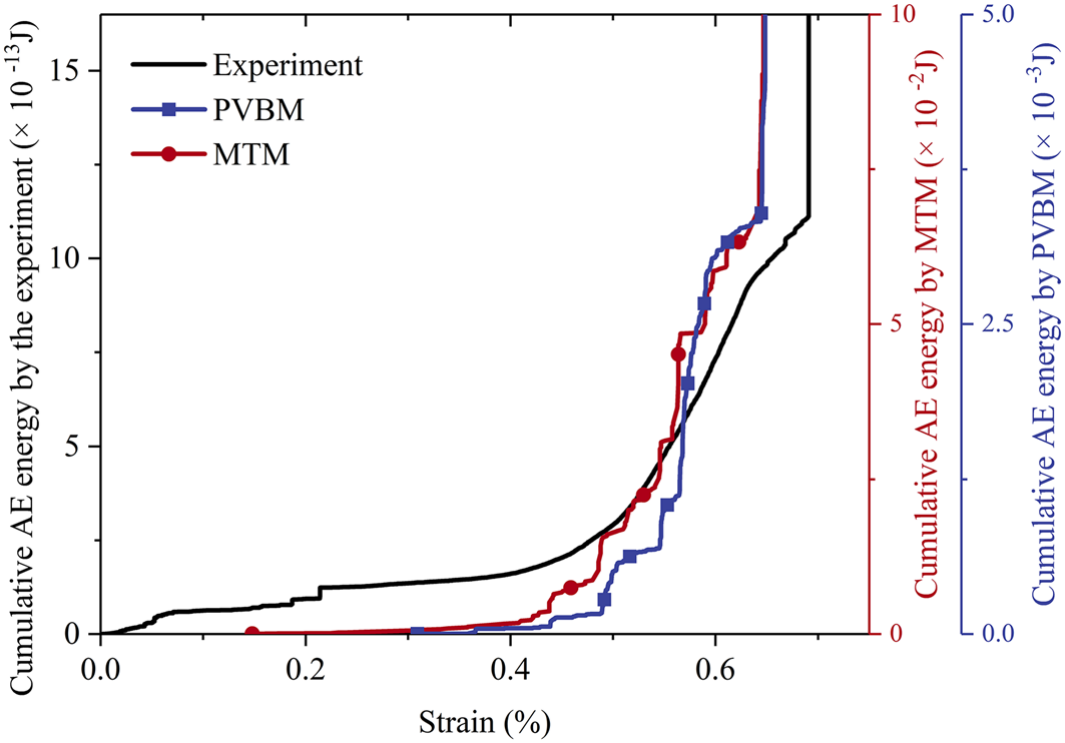
Comparison of cumulative energy by the experiment, PVBM and MTM.

After the evaluations of AE events, Fig. 13 shows the comparison of cumulative energy. The curves all show a similar variation trend in which cumulative energy remains approximately invariant at first, then increases greatly, finally increases sharply and instantly at the peak point. In addition, during the prepeak stages, both cumulative energy curves by PVBM and MTM are generated in a stepwise mode, and the corresponding thresholds are almost the same at strains of approximately 0.44%, 0.49%, 0.55%, 0.56%, 0.60% and 0.64%.

However, the orders of magnitude of AE energy are different. The AE energy by MTM is approximately 6 × 10^10^ times greater than the AE energy by the experiment, similar to the findings by Khazaei et al^62^. In addition, the AE energy by MTM is approximately 20 times greater than the AE energy by PVBM. It is worthwhile to investigate the latter difference.

An intuitive explanation is the attenuation of elastic waves in the form of damping in the DEM when using PVBM, which is necessary to reduce vibration by absorbing vibrational energy, manifested by the low damping leading to unnecessary continuous vibration, while high damping will lead to a decrease in AE amplitude and high frequency content^48^. The present example is a quasi-static simulation with a local damping coefficient *α* = 0.7, which has been proven to be high enough to prevent the formation of dynamic waves but insensitive to calculate AE energy by MTM^63^. Apart from the damping effect, radiated strain energy may be dissipated to friction at contacts and transfer to neighbour particles^24^. As a result, apparent energy (equivalently energy received at AE sensors in PVBM) may appear by far less than radiated energy (equivalently epicentre energy in MTM), which has proven that the former energy decreases with the specimen size whereas the latter one increases^64,65^.

There is a direct correlation between AE energy and the scale of the hazard. In practical applications, the actual AE test acquires energy after partial attenuation on the surface of the tested body, which is difficult to calibrate due to the invisibility of the fracturing events. The PVBM allows this calibration since the fracturing events are visible and analysable in numerical simulation. In addition, the MTM can explicitly calculate the epicentre energy, which is helpful for the evaluation of the scale of the potential hazard. To reduce potential losses most, a practical AE test is suggested to be combined with both PVBM and MTM.

### b-value

**Figure 14:**
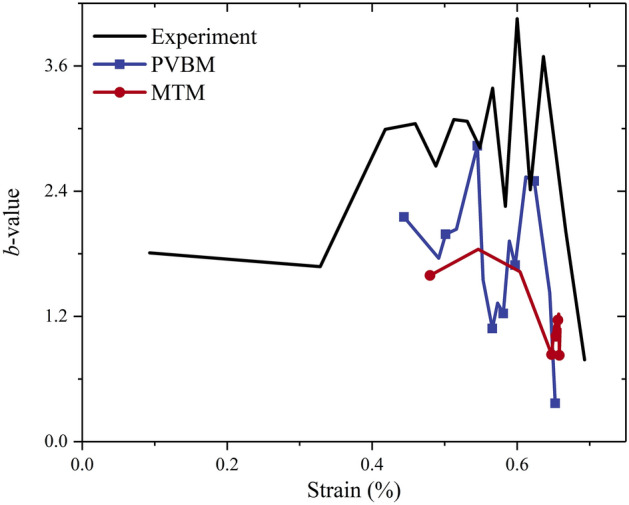
Comparison of *b*-value by the experiment, PVBM and MTM.

Figure 14 shows the comparison of the *b*-value. The three curves have a similar variation trend that first fluctuates, then decreases significantly at the peak points. However, the valid strain range to calculate the *b*-value and its magnitude fluctuation are different. For the difference in the valid strain range, the *b*-values are calculated from the strains of approximately 0.09%, 0.44% and 0.48% by the experiment, PVBM and MTM, respectively, since there is no AE event at the beginning of the simulation. For the difference in magnitude fluctuation, the lower magnitude fluctuation of the *b*-value by MTM resulted mainly from the narrower range of magnitudes according to a previous study^66^. Some researchers questioned the reliability of the *b*-values because they were calculated in a statistical method by Eq. (5) to describe the AE amplitude distribution and were susceptible to the subjectivity of the researcher, such as the selection of the number of AE events, AE event segmentation and AE amplitude range^67^. For the MTM, the variation in the number of AE events is consistent with the variation in the number of cracks because cracks close in time and space are treated as a single AE event. It is natural to connect AE events with the number of bonds, which is directly related to the particle size. Khazaei et al.^41^ also pointed out that the number of AE events was a function of model resolution and found that coarser particles would result in smaller *b*-values.

The *b*-value has been proven to be able to identify states of damage despite the limitation in accurately evaluating the degree of damage^40^. As shown in Fig. 14, the *b*-value curves of both the PVBM and MTM increase when the strain falls into the range (0.49%-0.55%), corresponding to the formation of new cracks and restriction of crack propagation. Then, they decrease at a strain of approximately 0.55%, which indicates fracturing events are on a relatively large scale. However, there remains a difference in which the subsequent *b*-value by PVBM increases but the *b*-value by MTM decreases continuously, which would be explained by multiple reflections of elastic waves induced by previous fracturing events on a large scale. Finally, the *b*-values by both MTM and PVBM decrease at the related peak point.

The *b*-value is always adopted to characterize the scale of the AE magnitude distribution. The identification of damage states by the *b*-value plays an important role in the prevention of rock mass engineering disasters. The above comparison indicates that the errors of experimental *b*-values resulting from undesirable noise in the actual AE test can be removed from numerical simulation. Considering both reliable prevention and low cost in engineering practice, the actual AE test should be combined with PVBM and MTM to accurately identify damage states.

### Consistency and compatibility

As a proxy model, PVBM is highly consistent with the experiment in principle and process, which is theoretically available at the 3D level. In addition, the PVBM is compatible with the MTM since both are implemented in the same numerical model. Comparisons indicate that AE characterization by PVBM shows consistent variation trends with the AE characterization by the experiment and MTM, in particular, AE events by PVBM are closer to the AE events by the experiment, and the stepwise mode of the cumulative energy curve by PVBM is closer to the stepwise mode of the cumulative energy curve by MTM. Furthermore, compared with MTM, PVBM has the advantages of a smaller computation amount and easier programming, reflected by an approximately 5 times higher calculation speed in the present simulation. However, PVBM is not as accurate as MTM since it inherits some limitations from experiments, such as reflection and attenuation of elastic waves. Hence, PVBM may be used as an alternative model in qualitative AE characterization for some very complex problems, such as particle-intensive models and special geometrical structures.

Although MTM is not compatible with the experiment in principle and process, the feasibility was verified via PVBM. Comparisons indicate that AE characterization by MTM shows high consistency with PVBM in AE energy. In addition, MTM is of great accuracy since MTM is not involved in the influence of reflection and attenuation of elastic waves, which indicates that MTM is applicable to the ideal case. However, there is no perfect model^68^. MTM has high time consumption, especially for a 3D fine model with small particles.

### New insights into future AE characterization and applications

Currently, AE characterization by experiments and numerical simulations has been widely used in the protection and prevention of rock mass engineering disasters. For instance, earthquake prediction involves studying fault nucleation and growth using AE tests^14^ and simulations^69^, landslide real-time warning systems based on AE techniques^70–72^ and simulations^73^ and stability evaluations of tunnel excavations by monitoring seismic signals and AE simulations^57^. According to the current study, AE tests are realistic but constrained by attenuation and reflection of elastic waves. Compared with the experiment, the MTM can calculate the epicentre energy more explicitly, while it is difficult to appreciably identify the prepeak fracturing. Nevertheless, this limitation of MTM can be made up by PVBM, which is a proxy model to imitate the experimental process and is not as accurate as MTM. In addition, both the PVBM and MTM can reduce errors in the identification of damage states. Therefore, we can acquire new insights into future AE characterization and applications: Engineers may acquire AE characterization more reasonably and accurately by combining the advantages of AE experiments, PVBM and MTM, which provides improvements in the application of the AE technique.

## Conclusions

Comparison of AE characterization between MTM and the experiment illustrated that there were some remarkable discrepancies between them, including principle, processing method and energy analysis, as shown in Fig. 1. To fill in these gaps, this paper proposed a proxy named PVBM, achieved by directly monitoring and analysing the particle velocity in the numerical model, to provide a reasonable evaluation of AE characterization from the experiment to numerical simulation. Results revealed that the AE characteristics acquired by the experiment, PVBM and MTM, including AE event, energy and *b*-value, showed similar variation patterns. Note that the AE experiment acquired actual AE characterization but had limitations such as attenuation and reflection of elastic waves; MTM accurately calculated the AE energy but could not appreciably characterize the variation trend of AE events during the prepeak stages, which indicated the improvement in the evaluation on the hazard scale but the instability in the identification of hazard precursors; With good robustness, PVBM was consistent with the experiment in principle and process and compatible with MTM, which resulted that AE events by PVBM were closer to the AE events by the experiment, while AE energy by PVBM showed excellent consistency at thresholds corresponding to the stepwise growth stage with the AE energy by MTM. Besides, both PVBM and MVM reduced experimental *b*-value errors in the identification of damage states. Thus, a systematic combination of the advantages of PVBM and MTM was suggested to effectively prevent rock mass engineering disasters in practical AE applications.

## Data Availability

The datasets generated during and/or analysed during the current study are available from the corresponding authors on a reasonable request.
